# Applications of intraoperative Duplex ultrasound in vascular surgery: a systematic review

**DOI:** 10.1186/s13089-021-00208-8

**Published:** 2021-02-19

**Authors:** Pasha Normahani, Bilal Khan, Viknesh Sounderajah, Sepideh Poushpas, Muzaffar Anwar, Usman Jaffer

**Affiliations:** 1 Imperial Vascular Unit, Imperial College Healthcare NHS Trust, London, UK; 2 Department of General Surgery, Kingston Hospital, London, UK; 3 St Marys Hospital, Level 2, Patterson Building, Paddington, W21NY UK

**Keywords:** Duplex ultrasound, Carotid artery endarterectomy, Lower limb revascularisation, Completion imaging

## Abstract

**Objective:**

This review aims to summarise the contemporary uses of intraoperative completion Duplex ultrasound (IODUS) for the assessment of lower extremity bypass surgery (LEB) and carotid artery endarterectomy (CEA).

**Methods:**

We performed a systematic literature search using the databases of MEDLINE. Eligible studies evaluated the use of IODUS during LEB or CEA.

**Results:**

We found 22 eligible studies; 16 considered the use of IODUS in CEA and 6 in LEB. There was considerable heterogeneity between studies in terms of intervention, outcome measures and follow-up. In the assessment of CEA, there is conflicting evidence regarding the benefits of completion imaging. However, analysis from the largest study suggests a modest reduction in adjusted risk of stroke/mortality when using IODUS selectively (RR 0.74, CI 0.63–0.88, p = 0.001). Evidence also suggests that uncorrected residual flow abnormalities detected on IODUS are associated with higher rates of restenosis (range 2.1% to 20%). In the assessment of LEB, we found a paucity of evidence when considering the benefit of IODUS on patency rates or when considering its utility as compared to other imaging modalities. However, the available evidence suggests higher rates of thrombosis or secondary intervention in grafts with uncorrected residual flow abnormalities (up to 36% at 3 months).

**Conclusions:**

IODUS can be used to detect defects in both CEA and LEB procedures. However, there is a need for more robust prospective studies to determine the best scanning strategy, criteria for intervention and the impact on clinical outcomes.

## Introduction

Despite recent advances in the provision of enhanced risk factor modification strategies and personalised post-operative patient care, open arterial surgery remains a risky endeavour. In addition to its technical complexity, it is a practice that harbours, in relative terms, a high degree of morbidity and mortality.

In elective infra-inguinal arterial, lower extremity bypass surgery (LEB), early post-operative graft failure, can occur in up to 5% of cases [1], requiring further surgical intervention, and increased length of hospital stay. For carotid artery endarterectomy (CEA), there has been reported, 7% peri-operative risk of stroke/mortality in patients with symptomatic carotid artery disease [2].

Although the aetiology of such early complications is often multifactorial, it is estimated that up to 25% are caused by technical errors and are thus preventable [3–5]. To minimise preventable technical errors, intraoperative assessments of technical adequacy may be useful. Intraoperative assessments aim to identify technical problems that may need to be immediately revised. Visual inspection, palpation and continuous-wave Doppler assessment are limited by subjectivity. In contrast, completion angiography objectively evaluates technical adequacy and arterial run-off. However, complications of arterial puncture, the use of nephrotoxic contrast agents, time taken to perform and radiation exposure limit its use.

Duplex ultrasonography (DUS) incorporates both B-mode ultrasound and pulsed-wave Doppler to allow for non-invasive anatomical imaging as well as assessment of flow through colour Doppler, and qualitative assessment of graphically displayed waveforms. Although possibly less anatomically precise compared to angiography, it can identify defects in arterial anastomoses and can also identify low velocity flow which may be undetected by angiography.

This review aims to summarise the effectiveness of intraoperative completion DUS (IODUS) for the assessment of CEA and LEB.

## Methods

### Search strategy

Following Preferred Reporting Items for Systematic Reviews and Meta-Analyses (PRISMA) recommendations, an electronic database search was conducted using MEDLINE to include articles from January 1950 through to February 2020 written in English. Reference lists were examined from the retrieved full-text articles. ClinicalTrials.gov was searched for in-progress trials.

In our search strategy, we used the following key terms: “ultrasonography”, “Doppler”, “duplex”, “completion imaging”, “vascular surgical procedures”, “bypass grafting”, “lower limb arterial bypass”, “carotid artery endarterectomy” and “intra-operative”. Titles and abstracts were reviewed for relevance by two investigators (PN and BK) independently. Conference abstracts and protocol papers were not included. Full-text articles were then reviewed, and data collected on technique used, participants, interventions performed, outcomes and findings. Disagreements were resolved by consensus discussion with the senior author (UJ).

### Eligibility criteria

We sought studies that evaluated the use of IODUS during LEB or CEA. Restrictions were not placed on study type. However, studies only considering the natural progression of lesions (i.e., results not used to inform management decisions peri-operatively) were excluded.

### Outcomes measured

For the use of IODUS for CEA, outcomes of interest included (1) stroke/mortality at 30 days and (2) flow abnormalities on follow-up imaging. For LEB, the outcome of interest was primary graft patency at 30 days. For both CEA and LEB, we also consider the natural history of cases with normal and abnormal completion imaging.

## Results

**Fig. 1:**
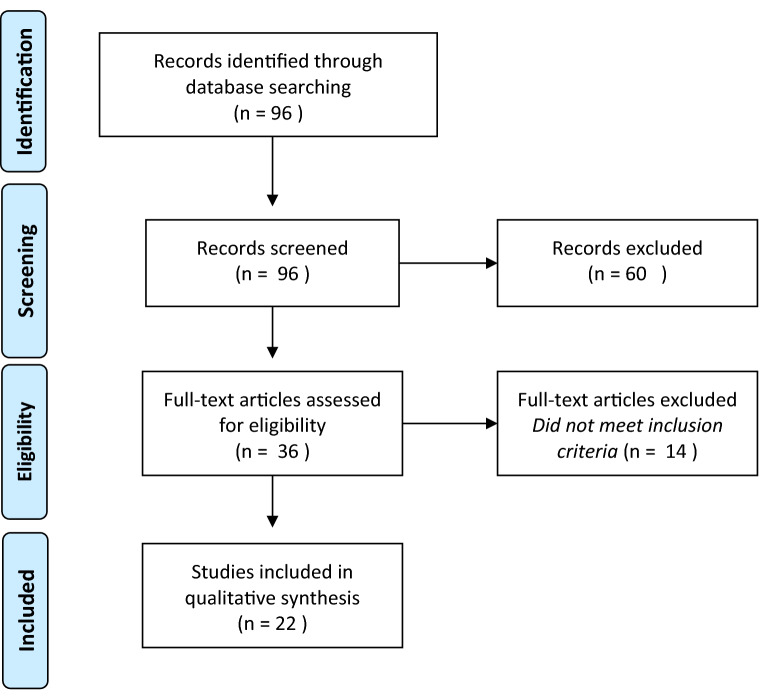
PRISMA systematic review flow diagram

Through our initial search strategy, we identified 96 papers (Fig. 1). Of these, 36 papers were shortlisted for full-text review based on their title and abstract. A full-text screening resulted in a final selection of 22 studies. Of these studies, 16 considered the use of IODUS in CEA (Table 1) and 6 in LEB (Table 2).

**Table 1:** Summary of results from studies evaluating IODUS in CEA

Carotid endarterectomy								
First author	Year	Study design	No of procedures	Imaging criteria (selective or routine)/revision criteria	Revision rate	Stroke (30 days)	Mortality (30 days)	Follow-up imaging; residual or recurrent stenosis
*Studies comparing (a) completion imaging vs no imaging or (b) different completion imaging modalities*								
Knappich C	2017	Retrospective analysis of registry data; *German statutory nationwide quality assurance database*	142,074	Selective; Imaging in 66.9% (95 044/142 074) of cases. Mix of DUS/angiogram/flowmetry or other unspecified modalities. Differential count for imaging modalities not presented in data Criteria for revision: Surgeons discretion	*Not known*	Combined stroke/mortality rate Scan: 1.7% (1654/95,044), RR 0.86 (CI 0.80–0.93) No scan: 2% (957/47,030) Adjusted risk of any stroke or mortality Intraoperative completion study Angiogram: RR 0.8 (CI 0.71–0.9) p < 0.001 DUS*:* RR 0.74 (CI 0.63–0.88) p = 0.001 Flowmetry*:* RR 0.87 (CI 0.74–1.04) p = 0.121 Other*:* RR 0.97 (CI 0.80–1.17) p = 0.756		*Not known*
Wallaert JB	2011	Retrospective analysis of registry data; *Vascular Study Group of New England (VSGNE) Registry*	6115	Selective; Completion imaging only performed in 2033 cases. DUS in 94% (1919/2033) of cases Amongst 73 surgeons; completion imaging used rarely (51%), selectively (22%) or routinely (27%) Criteria for revision: Surgeon’s discretion Practice pattern defined as routine (imaging used in ≥ 90% of cases), selective (5% to 90%) and rare (< 5%)	Routine: 7.6% Selective: 0.8%, Rare: 0.9%	Combined stroke/mortality rates Routine: 2.4% (42/1763) Selective: 1.2% (12/1018) Rare: 1.7% (55/3334) *Routine v selective *v* rare; p* = *0.048* *Risk adjusted; Selective (OR, 0.75; 95% CI 0.40–1.41; p* = *.366), routine OR, 1.42; 95% CI .93–2.17; p* = *.106* Revised cases: 3.9% (7/178) Not revised cases: 1.7% (102/5937) *Revised v not revised; p* = *0.028* *Risk adjusted; OR 2.1; 95% CI, 0.9–5.0; p* = *.076* Completion imaging: 2.6% No completion imaging: 1.3% *Completion imaging v no completion imaging; p* < *0.001* *Risk adjusted; OR, 1.9; 95% CI, 1.2–2.7; P* = *.002*		Restenosis (> 70%) at 1 year Routine: 1.1% Selective: 1.1% Rare: 2.8% Routine v selective v rare; *p* = 0.09
Rockman CB et al	2007	Retrospective analysis of data from the *New York Carotid Artery Surgery (NYCAS) study*	9278	Selective; Imaging in 35.8% (3318/9278) Angiogram 5.4% (178/3318), continuous wave Doppler 70.3% (2331/3318), DUS 17.6% (585/3318), combination of angio ± Doppler or DUS 5.9% (196/3318) Criteria for revision: Surgeons discretion	*Not known*	Combined stroke/mortality rate No scan*:* 3.8% Any scan*:* 4.3% Angiogram*:* 5.2% Doppler*:* 4.3% DUS*:* 4.3% *No statistical significance when comparing ‘no scan’ with ‘any scan’ or when comparing individual modalities with ‘no scan’*		*Not known*
Kinney EV	1993	Prospective single center study	461	Selective; DUS in 410 cases Criteria for revision: Severe flow disturbance (PSV > 150 cm/s and spectral broadening) or occlusion in the CCA/ICA/ECA	6.3% (26/410)	Stroke rate Combined*: *2.2% (10/461) No scan*: *0% (0/51) *Normal scan*: *2.1% (7/337) *Abnormal scan*: *4.1% (3/73) Revisions*: *3.8% (1/26) (*at completion)	Mortality rate Combined*:* 2.6% (12/461) No scan: 0% (0/51) *Normal scan: 3.3% (11/337) * Abnormal scan: 1.4% (1/73)	Flow abnormality at 3 months (> 50% category): Combined: 2.4% (11/461) No scan: 9.8% (5/51) *No flow abnormality: 0.3% (1/337) *Residual flow disturbance: 6.8% (5/73)
Lingenfelter KA	1995	Prospective single center study	53	Routine; combination of hand-held Doppler, DUS and angiogram in all cases Criteria for revision: Surgeon’s discretion	11.3% (6/53)	Stroke rate Combined*:* 1.8% (1/53) DUS detected all 6 defects requiring revision. Audible Doppler assessment detected only 1 and DSA 4	Mortality rate Combined*:* 0% (0/53)	*Not known*
Lipski DA	1996	Retrospective study	86	Selective (at surgeons discretion); DUS in 39 procedures Criteria for revision: Surgeon’s discretion	23.1% (9/39)	Stroke rate Combined*:* 3.5% (3/86*)* Scan: 2.6% (1/39) No scan: 4.3% (2/47)	Mortality rate Combined*:* 0% (0/86)	Residual stenosis (> 50%): Combined*:* 8.1% (7/86*)* Scan*:* 0% (0/39) No scan: 14.9% (7/47) Restenosis (< 50%) at mean follow up of 20 months Combined*:* 4.7% (4/86*)* Scan*:* 5.1% (2/39) No scan*:* 4.3% (2/47) *Note: significant variation in patch vs primary closure*
Lane RJ	1987	Prospective single center study	380	Selective; DUS in 175 cases Criteria for revision: Unspecified criteria for 30% stenosis	6.9% (12/175)	Stroke rate Combined***:*** 2.1% (8/380) No scan*:* 2.4% (5/205) Normal scan*:* 2.2% (3/136) Abnormal scan*:* 0% (0/39)	Mortality rate Combined*:* 0.5% (2/380) No scan*: 0.4%* (1/205) Normal scan: 0.7% (1/136) Abnormal scan: 0% (0/39)	Restenosis at mean follow up of 22 months Combined: not available No scan: not available Abnormal scan: 6.3% (1/16) Restenosis at mean follow up of 16 months Normal scan: 9.2% (8/87) *Only 103 patients followed up
*Descriptive studies*								
Dorffner R	1997	Prospective single center study	50	Routine; DUS in all cases Criteria for revision: Surgeons’ discretion	18% (9/50)	Stroke rate Combined*:* 4% (2/50) *Normal scan: 0% (0/32) Abnormal scan but not revised: 20% (2/10) Revised: 0% (0/9) *Normal at completion	Mortality rate Combined*:* 0% (0/50)	Restenosis at mean follow up of 10 months Normal scan: 6.3% (2/32) Abnormal scan but not revised: 20% (2/10) Revised: 0% (0/9)
Mays BW	2000	Prospective single center study	100	Routine; DUS in all cases Criteria for revision: PSV > 150 cm/s and spectral broadening indicating severe flow disturbance in CCA, ICA or ECA. If no obvious cause for flow disturbance was identified then angiogram was performed prior to revision. In addition, flaps in distal ICA and defects > 2 mm in the CCA or bulb were revised in the presence of a PSV > 125 cm/s colour mosaic or loss of spectral window	21% (21/100)	Stroke rate Combined*: *1% (1/100)	Mortality rate Combined*: *1% (1/100)	At 6 weeks: Revisions: 1/21 showed an occluded ICA and 2/21 showed residual stenosis No abnormality on completion: 10/79 showed recurrent stenosis (16% to 49% category)
Yuan	2014	Prospective single surgeon series	285	Routine**;** DUS in all cases Criteria for revision: Visible ICA kinking with PSV ≥ 120 cm/s	3.9% (11/285)	Stroke rate Combined: 1.1% (3/285) Repaired ICA kinking: 0% (0/11) Unrepaired ICA kinking: 0% (0/16)	Mortality rate Combined: *0% (0/285)* Repaired ICA kinking: 0% (0/11) Unrepaired ICA kinking: 0% (0/16)	By 9–24 months: Combined: *1.8% (5/284)* Repaired ICA kinking: 9.1% (1/11) restenosis (60–79%), 9.1% (1/11) occlusion Unrepaired ICA kinking: 0% (3/15*) mild 0–40% stenosis (*one patient lost to f/u)
Baker	1994	Retrospective single center study	316	Selective; DUS in 283 cases Criteria for revision: Surgeon’s discretion	2.8% (9/316)	Stroke rate Combined: 1.6% (5/316) Normal scan: 1.6% (4/254) Unrepaired minor defects: 1.9% (1/53) Revised defects: 0% (0/9)	Mortality rate Combined*:* 0.3% (1/283) *(Single death was in the normal scan group)* *316 cases in 283 patients	Stenosis (> 75%) at mean follow up of 21.6 months: Combined: 5.7% (18/316) Normal scan: 3.2% (8/251*) Unrepaired minor defects: 17.3% (9/52*). One of these arteries later occluded Revised defects: 11.1% (1/9) *Note: restenosis was correlated with primary and patch closure (p* = *0.025)* *No of patent vessels in surviving patients
Panneton JM	2001	Retrospective single center study	155	Routine; DUS in all cases Criteria for revision: PSV > 125 cm/s and marked plaque/thrombus or large intimal flap/dissection in the CCA, IC or ECA	9% (14/155)	Stroke rate Combined: 1.9% (3/155) Normal scan: 1.1% (1/91) Minor defects: 0% (0/47) Revised major defects: 0% (0/14) Unrevised major defects: 66.7% (2/3)	Mortality rate Combined: 0.7% (1/149*) (1 death in patient with unrevised major defect) *155 cases in 149 patients	Restenosis at 6 months*: Normal scan: 1.1% (1/91) restenosis Minor defects: 2.1% (1/47) Revised major defects: 0% (0/14) *(Asymptomatic > *50%)*
Steinmetz OK	1998	Retrospective single surgeon series	100	Routine; DUS in all cases Criteria for revision: Intraluminal thrombosis or focal elevation of PSV > 120 cm/s and marked spectral broadening in either CCA, ICA or ECA	2% (2/100)	Stroke rate Combined: 2% (2/100)	Mortality rate Combined: 1% (1/100)	Abnormality at mean follow up of 9.2 months Combined: 13.6% (12/88) *(*< *50% stenosis in 6,* > *50% stenosis in 5, asymptomatic occlusion in 1)* *Follow up scans available in 88 patients
Ascher E	2004	Prospective single center study	650	Routine; DUS in all cases Criteria for revision: Mobile flap > 2 mm in ICA, flap > 3 mm in the CCA and technical defects causing > 30% luminal ICA stenosis	2.3% (15/650)	Stroke rate Combined: 0.3% (2/650) Revised: 0% (0/15)	Mortality rate Combined: 0.3% (2/590) *650 cases in 590 patients	Flow abnormality at 2 weeks: Combined: 3% (2/625*)—ICA occlusion in both cases *Revised*: 0% (0/15) Flow abnormality at 3 months: Revised: 0% (0/15) *625 f/u scan available for 650 patients
Mullenix PS	2003	Prospective single center study	100	Routine; DUS in all cases Criteria for revision: Surgeon’s discretion	7% (7/100)	Stroke rate Combined: 2% (2/100) *Both stroked occurred in cases with an abnormal completion scan that was left unrepaired. However, one stroke was contralateral and likely unrelated*	Mortality rate Combined*:* 0% (0/100)	Re-stenosis* at follow up (range 6–45 months) Combined: 10/100 3 of these regressed and 1 was high grade (> 80%) *Defined as > 50%
Bandyk DF	1994	Prospective single center study	368 in total, of which 210 were CEA’s	Routine; DUS in all cases Criteria for revision: Defect on B mode with a PSV > 150 cm/s in CCA, ICA or ECA	8.1% (17/210)	Combined: 0% (0/210)	*Not specified*	*Not specified*

**Table 2:** Summary of results from studies evaluating IODUS in LEB

Lower limb revascularisations						
First author	Year	Study design	No procedures	Imaging criteria (selective or routine)/revision criteria	Revision rate	Graft thrombosis/revision
*Studies comparing a) completion imaging vs no imaging or b) different completion imaging modalities*						
Taze-Woei T	2014	Retrospective analysis of registry data; *Vascular Study Group of New England (VSGNE) Registry*	2032	Selective: completion imaging performed in 67% of cases (1368/2032). Angiography performed in in 89% and DUS in 11% of cases Criteria for revision: Surgeons discretion		No breakdown results for completion imaging strategy (i.e., DUS and angiography) ***Selective vs routine completion imaging*** The surgeon’s strategy of performing routine *vs* selective CIM was not associated with primary graft patency at discharge (RR, 0.8; 95% CI 0.6–1.1; *p* = .31) and at 1-year follow-up (RR, 1.1; 95% CI 0.9–1.2; *p* = .56) ***Completion imaging vs no completion imaging*** In multivariate models, completion imaging was not associated with improved primary graft patency at discharge (OR, 1.1; 95% CI 0.7–1.7; *p* = .64) or at 1 year (OR, 0.9; 95% CI 0.7–1.2; *p* = .47)
Gilbertson JJ	1991	Prospective single center study	20	Routine. Blinded comparison of DUS, angiography and angioscopy Criteria for revision: Residual valve cusp: Doubling of PSV or marked spectral broadening compared with adjacent graft. PSV < 40 cm/s or > 150 cm/s Unligated side branch: B-mode or color flow image directed away from lumen Anastomotic stenosis: High velocity jet or turbulence	unknown	***Residual cusps (n = 9)*** Angiography: (2/9) Angioscopy: (9/9) DUS: (1/9) ***Unligated side branch (n = 32)*** Angiography: (14/32) Angioscopy: (21/32) DUS: (4/32) ***Anastomotic stenosis (n = 0)*** Angiography: (4/0), FP rate 20% Angioscopy: (0/0) DUS: (2/0), FP rate 10% Denominator = confirmed on exploration
*Descriptive studies*						
Bandyk DF	1994 *(series from 1990–1993)*	Prospective single center study	368 in total, of which 135 were bypasses	Routine *Completion arteriography was also performed in 81% of cases (110/135)* Criteria for revision: Severe (PSV > 180 cm/s with broadening or PSVR 2.5 to 4) or high grade (PSV > 300 cm/s or PSVR > 4) stenosis velocity spectra in the presence of anatomic lesion on DUS	14.1% (19/135)	Within 30 days Combined graft thrombosis: 0.7% (1/135) Combined secondary intervention:* 2.2% (*3/135) *Due to a lesion and low graft flow* Combined assisted patency: 100% (135/135) *All of the four cases had residual lesions on completion DUS. 60% (3/5) unrepaired vein graft lesions required revision. No patients with a normal completion scan required a secondary procedure during a minimum of 2-month follow-up* *Completion angiography did not demonstrate any additional significant lesions*
Bandyk DF	1996 *(series from 1991–1995)*	Prospective single center study	275	Routine Criteria for revision: Severe (PSV > 180 cm/s with broadening or PSVR 2.5 to 4) or high grade (PSV > 300 cm/s or PSVR > 4) stenosis velocity spectra in the presence of vessel lumen defect or narrowing Lesions associated with low graft flow Segments with borderline stenosis (125–180 cm/s) were rescanned after additional papaverine administration—if PSV increased to > 200 cm/s in a normal diameter vein or anastomosis revision was performed When increased velocities (> 180 cm/s) were measured in outflow tibial arteries but the velocity ratio was less than < 2.5, an angiogram was performed confirm patency beyond distal anastomosis	15.6% (43/275)	Within 30 days *Graft thrombosis* Overall: 1.1% (3/275) Unrepaired flow abnormality (PSV < 180 cm/s): 4% (1/25) *Secondary intervention* Unrepaired flow abnormality (PSV < 180 cm/s): 16% (4/25) *Assisted patency* Overall: 100% (275/275) *Mortality:* Overall: 1.1% 3/275 Between 30 and 90 days *Graft thrombosis* Normal imaging*: 0.4% (1/235) Unrepaired flow abnormality (PSV < 180 cm/s): 4% (1/25) *Secondary intervention* Normal imaging*: 2.6% (26/235) Unrepaired flow abnormality (PSV < 180 cm/s): 36% (9/25) Abnormal imaging after repair: 40% (6/15) *Overall graft thrombosis/secondary intervention in normal imaging vs* Unrepaired *flow abnormality; p* < *0.001* *60% 15/25 uncorrected abnormalities had thrombosis or re-intervention in first 3 months* *202 without repair, 33 with repair
Johnson BL	2000 *(series from 1991–1998)*	Retrospective single center study		Routine Criteria for revision: Same criteria as Bandyk (1996). However, low flow and unrepaired graft lesions were managed with specified antithrombotic regimen or adjunctive procedure Normal graft flow (> 45 cm/s, low PVR defined as antegrade flow throughout pulsed cycle)- dextran + aspirin (325 mg/day) Low flow and low PVR- heparin + dextran + aspirin Low flow and high PVR- adjunct procedure (e.g., arteriovenous fistula or jump graft to another outflow artery) if possible. If not treated as low flow + low PVR Low flow graft stenosis (> 200 cm/s at site of stenosis- repair stenosis + heparin + dextran + aspirin	15.3% (96/626)* *Revision* of 99 graft *segments* for stenosis and 5 *adjunct procedure*s to improve graft flow * *104 defects in 96 bypasses	Within 30 days (secondary intervention rate) Combined: 4.2% (26/626) Normal flow: 1.1% (5/464) Normal flow (revised graft): 1.5% (1/67) Residual flow abnormality (revised graft): 34.5%(10/29) Unrepaired flow abnormality): 13.2% (7/53) Low flow (but no stenosis): 23.1% (3/13) Between 30 and 90 days (secondary intervention rate) Combined: 4.3% (27/626) Normal flow: 1.3% (6/464) Normal flow (revised graft): 1.5% (1/67) Residual flow abnormality (revised graft): 10.3% (3/29) Unrepaired flow abnormality: 24.5% (13/53) Low flow (but no stenosis): 15.4% (2/13) Total (within 90 days) (secondary intervention rate) Combined: 8% (51/626) Normal flow: 2.4% (11/464) Normal flow (revised graft): 3%(2/67) Residual flow abnormality (revised graft): 44.8% (13/29) Unrepaired flow abnormality: 37.7% (20/53) Low flow (but no stenosis): 38.5% (5/13)
MacKenzie KS	1999	Retrospective single center study	78	Selective Criteria for revision: Surgeons discretion	15.3% (12/78)	Within 30 days (n = 76): *Secondary intervention rate/primary patency/secondary patency* Normal flow: 1.3%/100%/*N.A* *Revised graft: 8.3%/100%/*N.A* Unrepaired flow abnormality: 11.1%/83%/*unknown* Between 30 days and an average intermediate follow-up of 7.4 months (n = 72) *Secondary intervention rate /primary patency/secondary patency* Normal flow: 1.3%/93%/97% Revised graft: 8.3%/91%/*100%* Unrepaired flow abnormality: 11.1%/53.1%/71.1% *Statistical significant difference in patency rates when comparing unrepaired flow abnormality to normal flow (p* < *0.001) or to repaired group (p* < *0.001)* *Repeat scan normal at completion in all 12 cases

### Quality of studies

There were no randomised controlled trials comparing IODUS with no completion imaging or other completion imaging techniques. Sixteen studies investigated the role of IODUS in CEA: 3 were based on prospectively maintained registries; 9 were prospective single-centre studies; and 4 retrospective studies. Six studies investigated the role of IODUS for LEB: 4 were prospective (in one study data was Registry analysis); 2 were retrospective studies. There was considerable heterogeneity in terms of intervention, outcome measures and follow-up.

### Carotid artery endarterectomy

#### Study characteristics and designs

IODUS was performed routinely in 9 of 16 studies and selectively in 7 of 16. Criteria for selective use of IODUS was left to surgeons’ discretion and was not specified in any of these studies.

Revision rates were available for 14 out of 16 studies and ranged between 0 and 23%. In 9 studies, the IODUS criteria for revision of the carotid reconstruction were unspecified and left to the discretion of the operating surgeon. In the remaining 7 studies, criteria for revision were variable depending upon the vessels scanned and were based-upon (1) spectral waveform criteria for flow disturbance and (2) B-mode criteria for determining significance of defects. In the majority of studies, all the three carotid vessels [6–10] were scanned. However, one paper only considered flow abnormalities in the presence of internal carotid artery (ICA) kinking [11] and another did not consider abnormalities of the external carotid artery (ECA) [12].

1. Spectral waveform criteria
  Criteria for defining severe flow abnormality were based on velocity readings (thresholds ranged from 120 to 150 cm/s) and qualitative arterial waveform features such as spectral broadening, colour mosaic and infilling of the spectral window. In three studies, vessels with flow abnormalities in the absence of an identifiable cause on B-mode ultrasound were surgically revised [7, 9, 10]. In two other studies, flow abnormalities with no identifiable cause on B-mode ultrasound were first assessed with an intraoperative angiogram prior to any revision [6, 12]. In one of these studies, an elevated peak systolic velocity (PSV) was measured in 27 cases (between 151 and 421 cm/s) with no evident technical defect or residual disease. In such cases, repeated measurements 15 to 20 min later were often improved (between 62 and 199 cm/s). If values were persistently abnormal, an intraoperative angiogram was then obtained.
2. B-mode criteria
  B-mode thresholds for revision were also variable and included the presence of ICA kinking, occlusion, thrombus, marked residual plaque, dissection or flap. Thresholds for determining the significance of flaps were also variable. Mays et al. [6] immediately revised all distal ICA flaps, and common carotid artery (CCA) or bulb defects > 2 mm in the presence of flow abnormalities. Panneton et al. [8] revised cases with intimal flaps or dissections > 3 mm in the presence of significant flow abnormalities. Ascher and colleagues [12] revised all cases with mobile flaps > 2 mm in ICA or > 3 mm in the CCA.

#### Comparing outcomes from completion imaging vs no completion imaging

The largest studies comparing utilisation of completion imaging vs no completion imaging are the retrospective analysis of large data sets from Knappich [13], Wallaert [14] and Rockman [15].

In the largest of these data sets, Knappich [13] demonstrated an association between completion imaging with lower rates of stroke/mortality (relative risk (RR) 0.86 (CI 0.80–0.93)). Rockman and colleagues, on the other hand, demonstrate no statistically significant difference in the rates of stroke [2.8% with imaging, 2.4% without imaging, *p* = not significant (NS)] or combined stroke/mortality rates (3.6% with imaging, 3.3% without imaging, *p* = NS) between cases in which intraoperative imaging was used and cases in which no intraoperative imaging was used [15]. Conversely, Wallaert and colleagues demonstrated higher rates of stroke/mortality in cases in which completion imaging was used as compared to cases in which no imaging was used (2.6% with imaging, 1.3% with no imaging; *p* < 0.001) [14]. This difference was still statistically significant after risk adjustment [odds-ratio (OR), 1.9; 95% CI 1.2–2.7; p = 0.002] [14].

#### Comparing outcomes from IODUS with other completion imaging modalities

Knappich et al. provide an analysis of 142,074 CEAs from the German statutory nationwide quality assurance database [13]*.* Within this large cohort, 66.9% (95,044) underwent completion imaging using IODUS, angiogram, flowmetry or other. In their results, they provide subgroup analysis demonstrating that utilisation of either intraoperative angiography (RR 0.8 (CI 0.71–0.9) *p* < 0.001) or IODUS (RR 0.74 (CI 0.63–0.88) *p* = 0.001) is associated with lower rates of stroke/mortality. Their analysis seems to show a slightly stronger affect for IODUS as compared to angiography.

Another study by Rockman and colleagues provides analysis from 9278 CEAs from the New York Carotid Artery Surgery (NYCAS) study [15]*.* Amongst these cases, completion imaging was performed in 3318 cases. In the majority of cases, imaging merely consisted of continuous wave Doppler assessment (70.3%; 2331/3318), followed by IODUS (17.6%; 585/3318), angiogram (5.4%; 178/3318), or a combination of angiogram ± Doppler or IODUS (5.9%; 196/3318). Stroke/mortality rates for each modality were not statistically significant (angiogram: 5.2%, Doppler: 4.3%, IODUS: 4.3%; *p* value not given).

A smaller prospective study of 53 patients compared the ability to detect abnormalities with audible handheld Doppler assessment, digital subtraction angiography (DSA) and IODUS colour flow [16]. In this cohort, 6 patients (11.3%) required revision due to significant abnormalities. IODUS detected all six defects requiring revision, whilst audible Doppler assessment detected only 1 and DSA 4 [16].

#### Primary revision surgery based on IODUS findings and 30-day stroke/mortality risk

Eight of the 16 studies presented data on stroke/mortality rates. The largest of these was a retrospective analysis of the Vascular Study Group of New England (VSGNE) Registry performed by Wallaert and colleagues [14]. In this studyWO completion imaging was performed in 2033 CEAs. The mainstay imaging modality of choice was IODUS (94% of cases; 1919/2033). They found the combined stroke/mortality rate was significantly higher in revised group as compared to cases not requiring revision (3.9% (7/178) v 1.7% (102/5937); *p* = 0.028). However, this was not statically significant after risk adjustment (OR 2.1 (CI 0.9–5.0); *p* = 0.076). Data regarding follow-up imaging for these two groups was not available for comparison.

The remaining 7 studies were either of too small sample size or did not include meaningful statistical comparisons of revised vs non-revised groups [7, 8, 11, 12, 17, 18]. Cumulatively, the stroke rates in these studies was 1% (1 of 101) in cases requiring revision and 1.2% (24 of 2026) in cases not requiring revision based on completion imaging.

#### Follow-up of revised cases

Six studies included data on follow-up imaging of revised cases [6, 8, 11, 12, 17, 18]. Cumulatively in these studies, abnormalities were detected in 7.6% of revised cases (6 of these 79 cases) and 2.6% (37/1448) of unrevised cases. However, meaningful comparison and interpretation of this data is challenging due to variable follow-up periods (2 weeks to 24 months), variable or unclear criteria for stenosis assessment, lack of risk adjustment and variable surgical techniques (e.g., patch plasty, primary closure, eversion). Of the 6 abnormalities described, 2 were of asymptomatic occlusions and 4 of stenosis.

#### Follow-up of ‘non-significant’ findings detected on IODUS

Six studies included descriptive analysis of stroke rates for cases in which abnormal completion imaging results were not considered significant and thus not revised [7, 8, 11, 17–19]. Cumulatively for these studies, stroke rates were 1.6% (18/1108) in cases with normal completion studies as compared to 2.5% (6/238) in cases with abnormal completion imaging.

Similarly, 5 studies included descriptive analysis of abnormalities detected on follow-up imaging [7, 8, 11, 17, 18]. Cumulatively for these studies, abnormalities on follow-up scans were detected in 1.2% (12/968) in cases with normal completion imaging as compared to 10.3% (19/185) in cases with abnormal completion imaging.

### Lower extremity bypass surgery

#### Study characteristics and designs

Six studies investigating the role of IODUS completion imaging in LEB were included. All studies considered infra-inguinal bypass procedures with vein conduit. Three of these studies are sequential publications from the University of South Florida group [10, 20, 21]. It is not made explicitly clear from the manuscripts whether each builds upon the previously published series, but this is implicitly suggested by the overlapping periods of data collection. Of the other studies, one compared completion imaging vs no completion imaging [22] and the other compared the accuracy of angiography, IODUS and angioscopy as completion imaging modalities [23]. IODUS was performed routinely in 4 of 6 [10, 20, 21, 23] studies and selectively in 2 of 5 [22, 24]. The decision to use selective IODUS was left to the discretion of individual surgeons. However, when used selectively, IODUS was performed mostly when the outflow artery was a tibial or tibioperoneal trunk [22]. Four out of 6 studies provided the revision rate, which ranged between 10 and 27% [10, 20–22].

Two studies did not include their criteria for intraoperative revision, which was left to the discretion of the operating surgeon [22, 24]. In the remaining studies criteria for defining severe flow abnormalities were based on peak systolic velocity reading of > 180 cm/s, grading of residual lesions (velocity ratio of > 2.5 was considered significant), and qualitative arterial waveform features including spectral broadening and absence of diastolic flow. If abnormalities were found, the hemodynamic response to flow augmentation was either evaluated by transverse imaging or rescanned after the administration of papaverine. Three studies assessed flow abnormalities with no identifiable cause on B-mode ultrasound followed by on table angiography before any revision [10, 20, 21]. In addition, if high velocities were identified in the outflow tibial arteries, with a velocity ratio of less than 2.5, then angiography was performed [10, 21].

#### Comparing outcomes from completion imaging vs no completion imaging

Only one study compared primary graft patency following infra-inguinal lower extremity bypass (LEB) between cases in which completion imaging was used vs those in which it was not [22]. In this retrospective analysis of registry data, completion imaging was used by 67.3% (*n* = 1368/2032) of vascular surgeons, with 67% using it selectively (< 80% of LEBs) and 33% routinely (≥ 80% of LEBs). The most commonly used imaging modality was angiography (89%, *n* = 1217/1368) followed by IODUS (11%, *n* = 151/1368). They authors found no association between using completion imaging and improved primary graft patency at discharge (OR, 1.1; *p* = 0.64) or at 1 year (OR, 0.9; *p* = 0.47), with similar results in bypass procedures performed with or without completion imaging. However, number of patients who had IODUS performed were comparatively much smaller (*n* = 151) as compared to patients who had angiography (*n* = 1217). Similarly, no effect was found between the surgeons’ strategy to perform completion imaging selectively or routinely on bypass graft patency at discharge (RR, 0.8; *p* = 0.31) or at 1 year (RR, 1.1; *p* = 0.56).

#### Comparing IODUS with other completion imaging modalities

Only one study compared IODUS against other modalities in lower extremity bypass procedures. However, this paper considered diagnostic accuracy and not clinical outcomes such as primary patency. Gilbertson conducted a prospective analysis of 20 femoral-infragenicular bypass procedures using in situ saphenous vein grafts [23]. They compared the ability to detect three specific abnormalities (patent vein side branches, residual valve cups and anastomotic stenoses > 30%) with angiography, angioscopy and IODUS. Within this cohort, 63 critical graft defects were identified by at least one of the imaging modalities and 41 of these were confirmed by direct inspection. Their results suggest that sensitivity of angioscopy (66% *n* = 21/32) and angiography (44%, *n* = 14/32) is higher than IODUS (12%, *n* = 4/32) for detecting patent vein branches (*p* < 0.01). For the detection of residual valve cups, angioscopy was the most sensitive (100%, *n* = 9/9), followed by angiography (22%, *n* = 2/9) and IODUS (11%, *n* = 1/9). They detected no anastomotic stenoses but false-positive rates were highest for angiography (20%), followed by IODUS (10%) and angioscopy (0%).

#### Follow-up of revised cases and those with ‘non-significant’ findings detected on IODUS

Johnson et al. [21] retrospectively identified 626 infrainguinal vein bypass procedures, where IODUS was used as the completion imaging. Of these, 15% (*n* = 96/626) were found to be abnormal, leading to the revision of 99 graft segments. The most commonly identified problem on imaging was the result of incomplete valve lysis (63%, 31/49). They found an improvement in the velocity spectra of 71% of segments and residual moderate stenosis in 29% of segments following graft revision. They found a significantly higher revision rate (27%, *p* < 0.01) with the use of alternative vein grafts as well as an increase in the frequency of unrepaired graft defects (*p* < 0.05). Johnson et al. found that secondary intervention rates within the first 90 days were highest for cases were there was an unrepaired flow abnormality as compared to those with a normal flow profile (37.7% vs 2.4%). Interestingly, in cases, where repair was performed, outcomes were considerably better if normal flow profile was established compared to if residual flow abnormality was detected (3% vs 44.8%) [21].

Another retrospective study by Bandyk et al. [20], considered 275 infrainguinal vein bypasses assessed using colour IODUS. A total of 50 (16%) abnormalities were detected in 43 grafts and necessitated revision. The revision rate was lowest for reversed saphenous vein bypasses (7%, *p* < 0.02) compared to other grafting techniques. Revision rate for popliteal and tibial bypasses were similar (14% vs 17%). Combined graft thrombosis and secondary revision rates at 90 days in those cases with normal completion imaging as compared to those with unrepaired flow abnormalities was significantly lower (graft thrombosis 0.4% vs 4%, secondary revision 2.6% vs 36%; combined *p* < 0.001). Overall, 15 out of 25 (60%) cases with uncorrected flow abnormalities had thrombosis or re-intervention in the first 3 months.

In a single centre retrospective study by MacKenzie et al. of 78 cases, secondary intervention rates at 30 days were lowest for cases with normal completion imaging (1.3%), followed by revised cases (8.3%) and unrepaired flow abnormalities (11.1%) [24]. They detected a statistically significant difference in patency rates when comparing unrepaired flow abnormality to normal flow (*p* < 0.001) or to repaired group (*p* < 0.001).

## Discussion

In this systematic review, we have summarised current evidence relating to the use of IODUS for CEA and LEB.

### Carotid artery endarterectomy

For completion assessment of CEA, there is conflicting evidence regarding the benefits of completion imaging from analysis of registry data [13–15]. However, the largest of these studies (over 140,000 cases) reports a modest reduction in adjusted risk of stroke/mortality when using IODUS selectively (RR 0.74, CI 0.63–0.88, *p* = 0.001) [13]. The results also suggest that outcomes when using IODUS are at least as good as intraoperative angiography. An opposing result reported by Wallaert and colleagues, suggests a higher stroke rate when using completion imaging (risk adjusted OR 1.9, CI 1.2–2.7, *p* = 0.002). However, when comparing different practice patterns, they found that the lowest rates were seen in cases, where completion imaging was used selectively (routine 2.4%, selective 1.2%, rare 1.7%; *p* = 0.048). This suggests that selective practice may be the most effective strategy, although the criteria for selecting cases was not explored in any of the studies. Wallaert and colleagues also noted that the rate of restenosis at 1-year follow-up was highest for cases, where completion imaging was rarely used (routine 1.1%, selective 1.1%, rare 2.8%; *p* = 0.09) [14]. This may be due to the failure to detect residual defects which may progress during the follow-up period. Data from other studies suggests that ‘non-significant’ residual defects detected on IODUS are associated with higher rates of restenosis during the follow-up (range 2.1% to 20%) [7, 8, 17–19]. These finding would suggests that although revision surgery can improve outcome, it is certainly not without risk and not all abnormalities detected on IODUS necessitate surgical revision. Isolated high velocities in the absence of other concerning waveform features, such as waveform broadening, or B-mode abnormalities may be related to vessel spasm [6]. If acted upon, these may add risk of complication. Parsa et al., have proposed protocolised imaging and interpretation guidance for both carotid and lower limb completion imaging [25].

### Lower extremity bypass surgery

There is paucity of evidence when considering the benefit of IODUS on patency rates following LEB. This may be because of perceived challenges in scanning smaller calibre vessels in a larger deeper surgical field. In the single study addressing IODUS, it was only used in 11% of cases [22], limiting its relevance.

A single paper comparing IODUS with other completion modalities. This study, by Gilbertson et al., compared angioscopy, angiography and IODUS and concluded that angioscopy and angiography were superior to IODUS in detecting residual cusps and un-ligated side branches. However, this study is also limited by its small sample size of 20 and was conducted almost 30 years ago.

Johnson and colleagues suggests that most benefit from IODUS scanning may be gained for in-situ and non-reversed translocated bypasses, as they have a significantly higher rate of lesions requiring revision [21]. Their results also report a 90-day secondary re-intervention rate of 37.7% in grafts with residual flow abnormalities. In comparison, grafts with normal flow, either without or following revision, revision rates of 2.4 and 3% respective, were reported. MacKenzie and colleagues report similarly, but with lower rates of secondary intervention in grafts with un-corrected flow abnormalities (11.1% within 7 months). This may have bearing on optimal post-operative surveillance strategy.

## Limitations

None of the studies were of randomised controlled trial design. There was also considerable heterogeneity between studies in terms of intervention, outcome measures and follow-up. Therefore, it was not possible to perform a meta-analysis.

### Future work

There is a need for well-designed prospective, multicentre randomised controlled trials to evaluate the effectiveness of IODUS in comparison to other modalities in reducing stroke/mortality outcomes in CEA procedures and primary patency in LEB. Further data are also required to determine the natural progression of different defects detected on IODUS to achieve evidence-based consensus on criteria for revision surgery.

## Conclusion

IODUS is a sensitive method to detect defects in both CEA and LEB. However, there is a need for more robust prospective studies to determine the best scanning strategy, criteria for intervention and the impact on clinical outcomes.

## Data Availability

Not applicable.
